# Incremental Prognostic Value of Albumin-Anchored Inflammatory Ratios Beyond a SOFA-Based Model in a Medical ICU: A Retrospective Cohort Study

**DOI:** 10.3390/metabo16090692

**Published:** 2026-09-18

**Authors:** Özkul Yılmaz Çolak, Melda İşevi, Tuğçehan Sezer Akman, Özgür Kılıç, Neslihan Ünal Akdemir

**Affiliations:** 1 Division of Intensive Care, Department of Internal Medicine, Faculty of Medicine, Ondokuz Mayıs University, 55139 Samsun, Türkiye; ozgur.kilic@omu.edu.tr; 2 Division of Intensive Care, Department of Anesthesiology and Reanimation, Faculty of Medicine, Ondokuz Mayıs University, 55139 Samsun, Türkiye; drmeldaisevi@gmail.com (M.İ.); tgchnszr@gmail.com (T.S.A.); neslihan.un.ak@gmail.com (N.Ü.A.)

**Keywords:** C-reactive protein-to-albumin ratio, procalcitonin-to-albumin ratio, intensive care unit, mortality, risk stratification, SOFA, net reclassification improvement

## Abstract

**Background/Objectives:** We compared three albumin-anchored ratios calculated from measurements obtained during the first 24 h, C-reactive protein (CRP)-to-albumin (CAR), procalcitonin-to-albumin (PAR) and lactate-to-albumin (LAR), as predictors of intensive care unit (ICU) mortality beyond an established severity model. **Methods:** Eligibility required an ICU stay reaching 24 h and a complete early work-up; 372 of 1352 screened admissions qualified, with each ratio using the worst component values in that window. Incremental value over a base model (Sequential Organ Failure Assessment (SOFA) score, sex, age-adjusted Charlson index) was assessed by change in the area under the curve (AUC; DeLong test), reclassification metrics (NRI, IDI) and calibration, with alternative baselines and outcomes, multiplicity adjustment, and selection weights from the excluded admissions. **Results:** Of 372 patients, 248 (66.7%) died. As standalone predictors, CAR (0.671), PAR (0.689) and LAR (0.642) were indistinguishable and inferior to SOFA (0.730). Only CAR significantly improved AUC discrimination (ΔAUC +0.030, *p* = 0.024; NRI +0.39; IDI +0.042); PAR and LAR did not significantly improve AUC discrimination. Entered together, CAR remained independent (aOR 1.52, 1.15–2.03), while PAR did not (1.20, 0.89–1.63). The cohort was sicker than the 626 patients excluded for incomplete work-up; after weighting, CAR stayed independent (aOR 1.47) but ΔAUC fell to +0.018. The increment was significant against SOFA alone (+0.033) but not against baselines including admission diagnosis or organ support; it remained significant after Benjamini–Hochberg but not Bonferroni adjustment. With in-hospital mortality it was larger (+0.044), but PAR then also retained independence. **Conclusions:** The first 24 h CAR provided a well calibrated improvement beyond a parsimonious SOFA-based model, but the increment was small and not robust to richer baselines or to multiplicity correction, and no subgroup-specific claim is supported. These hypothesis-generating findings need prospective validation.

## 1. Introduction

Accurate risk stratification during the first day of intensive care unit (ICU) care underpins triage, resource allocation, goals-of-care discussions, and the design and interpretation of clinical trials. Validated severity scores such as the Sequential Organ Failure Assessment (SOFA) quantify the degree of acute organ dysfunction and are strongly and reproducibly associated with mortality [1,2]. However, such scores require the aggregation of multiple physiological and laboratory variables and do not directly capture the host inflammatory and nutritional state that powerfully modulates the outcome in critical illness. Simple, inexpensive markers that can be obtained from bloodwork performed routinely in the first hours of ICU care and that complement, rather than replace, established scores therefore remain of considerable practical interest.

Serum albumin is both a negative acute-phase reactant and an index of physiological and nutritional reserve, and hypoalbuminaemia is consistently associated with adverse outcomes in acute illness. Composite ratios placing an inflammatory or perfusion marker over albumin have therefore attracted attention, since the denominator amplifies the prognostic signal of the numerator while remaining derivable from routine early tests. The C-reactive protein (CRP)-to-albumin ratio (CAR) is the best studied, predicting mortality in postoperative ICU patients and in unselected hospitalised older adults independently of admission diagnosis and often discriminating better than CRP or albumin alone [3,4]; the related lactate-to-albumin ratio (LAR) has likewise emerged as a robust prognostic marker in critical illness [5].

By comparison, the procalcitonin-to-albumin ratio (PAR) has received considerably less attention, and the available evidence is inconsistent. PAR has shown prognostic value in selected settings, including post-cardiac-arrest syndrome, where procalcitonin reflects the magnitude of the systemic bacterial inflammatory response [6]. Yet in a recent tertiary cardiac-ICU cohort that evaluated all three albumin-anchored ratios simultaneously, LAR and CAR each remained independently associated with ICU mortality after adjustment, whereas PAR did not [5]. Direct, head-to-head comparisons of CAR and PAR in adults are scarce and have largely been confined to selected populations such as the emergency department, post-cardiac-arrest care, or cardiac intensive care, rather than a general, mixed medical ICU.

Two further gaps are notable. First, most studies have tested only whether a ratio is associated with mortality, or have compared its crude discrimination, rather than asking whether it adds value beyond an established severity baseline; reclassification metrics such as the net reclassification improvement (NRI) and the integrated discrimination improvement (IDI) are seldom reported. Second, the comparative behaviour of CAR and PAR within clinically meaningful subgroups, particularly septic versus non-septic admissions in which the inflammatory numerators may behave very differently, has rarely been examined. We therefore conducted a retrospective cohort study in a high-acuity medical ICU to evaluate (i) whether first 24 h CAR and PAR provide discriminative and reclassification value for ICU mortality beyond a reference model based on SOFA, sex and the age-adjusted Charlson Comorbidity Index; (ii) how the two ratios compare with each other, both as standalone predictors and when entered together; and (iii) whether their prognostic performance differs between septic and non-septic admissions. Because both the SOFA score and each ratio are defined on the worst component values observed across a full 24 h period, the study was designed as a 24 h landmark analysis, and the population of interest is therefore patients still in the ICU one day after admission.

## 2. Materials and Methods

### 2.1. Study Design and Setting

This was a single-centre, retrospective observational cohort study, reported in accordance with the Strengthening the Reporting of Observational Studies in Epidemiology (STROBE) statement [7]. The study was conducted in the medical (internal-medicine) ICU of a tertiary-care teaching hospital in Samsun, Türkiye. Consecutive adults admitted to the unit between 1 June 2025 and 1 June 2026 were screened for eligibility. The study was conducted in accordance with the Declaration of Helsinki and was approved by the Clinical Research Ethics Committee of Ondokuz Mayıs University (protocol code 2026/394; date of approval 15 June 2026). Because the analysis was based on anonymised, routinely collected data, the requirement for informed consent was waived by the committee. The chronology of approval was as follows. The original protocol, covering the retrospective analysis of the eligible cohort, was approved on 15 June 2026, and data collection and analysis for that cohort were completed under it. After the analysis was complete, the extent of cohort selection was identified as the principal limitation of the work, and an application to extend the same protocol was submitted to the committee. The extension, which permits retrieval of a limited set of demographic, severity, organ-support and outcome variables for admissions that did not meet the eligibility criteria, was approved before any of those data were accessed. No laboratory values were requested, no additional patient contact or intervention was involved, and all data remained anonymised throughout. The study followed a landmark design: the exposure window was the first 24 h of ICU admission and the outcome was assessed from the end of that window onwards. The population of interest was therefore adults who were still in the ICU 24 h after admission and in whom a complete early inflammatory and metabolic work-up had been obtained, and all estimates reported here are conditional on that landmark and on that requirement.

### 2.2. Participants

**Figure 1:**
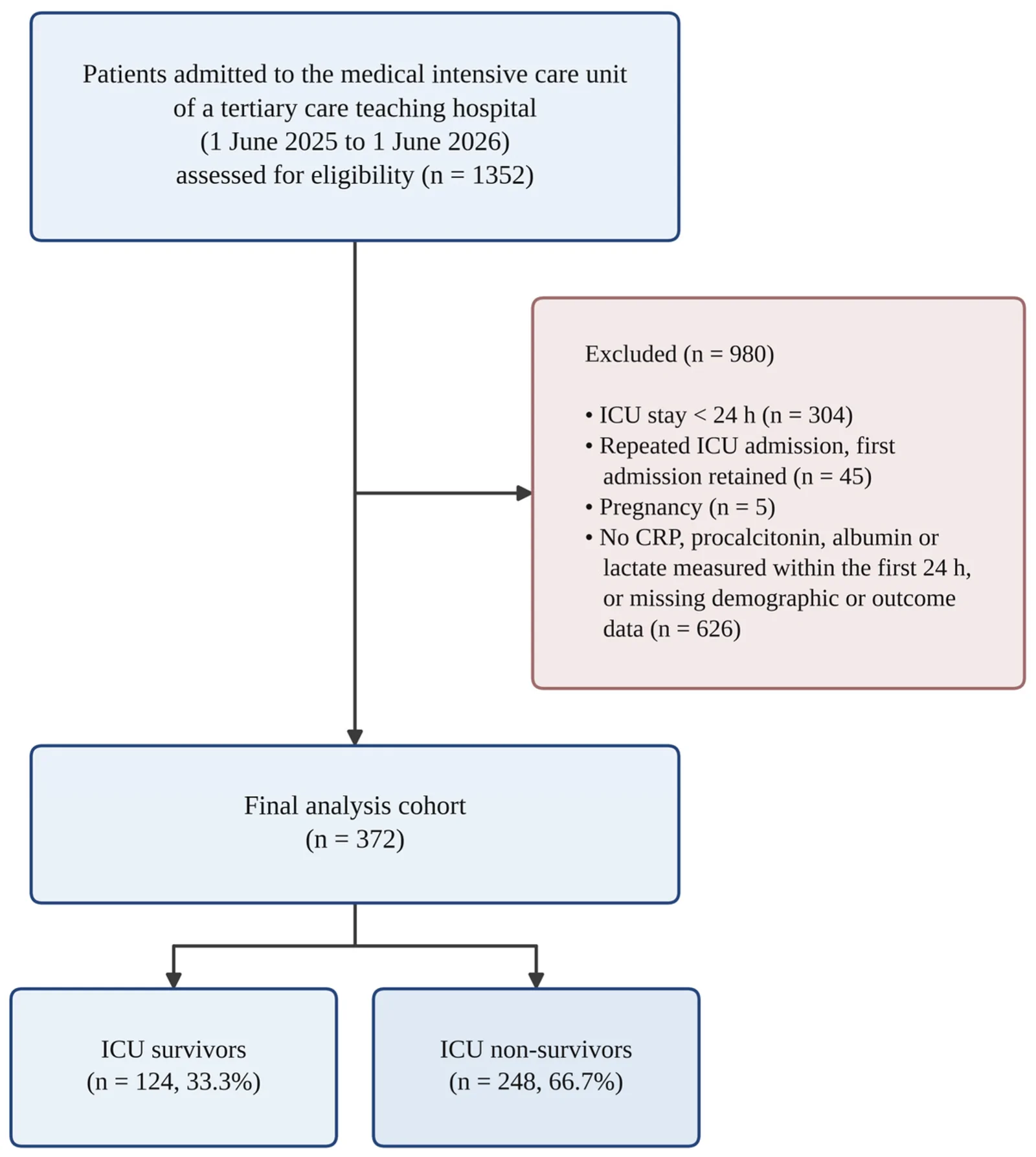
Study flow diagram of patient screening, exclusions and the final analysis cohort. ICU, intensive care unit.

Adults aged 18 years or older were eligible if they were admitted to the ICU during the study period, completed a full 24 h observation window in the unit, and had CRP, procalcitonin, albumin and lactate all measured within that window, with complete clinical and outcome data available in the hospital information system. For patients with more than one ICU admission during the period, only the first admission was analysed. Patients were not eligible if they were younger than 18 years, did not complete the 24 h observation window (ICU stay shorter than 24 h), were pregnant, had any of the four index laboratory values missing, were repeat (non-index) admissions, or had missing demographic or outcome data. Of 1352 admissions screened, 980 were excluded (ICU stay < 24 h, *n* = 304; repeated admission with only the first retained, *n* = 45; pregnancy, *n* = 5; missing index laboratory, demographic or outcome data, *n* = 626), leaving 372 patients in the final analysis cohort (Figure 1). The 24 h observation window was a measurement requirement rather than an outcome-based filter: the SOFA score and each of the three ratios are defined on the worst component values recorded across a full 24 h period, so neither can be computed in a comparable way for a shorter stay. The criterion was pre-specified, was applied uniformly to every consecutive admission, and was independent of biomarker values and of the outcome.

### 2.3. Variables and Definitions

The primary outcome was all-cause ICU mortality. The exposures of interest were three albumin-anchored ratios from the first 24 h, with each calculated from the worst (most abnormal) value of its components recorded within the first 24 h of ICU admission, in keeping with the SOFA scoring window: the CRP-to-albumin ratio, CAR = CRP (mg/L)/albumin (g/dL); the procalcitonin-to-albumin ratio, PAR = procalcitonin (ng/mL)/albumin (g/dL); and the lactate-to-albumin ratio, LAR = lactate (mmol/L)/albumin (g/dL). Because each component was selected as the worst value within the window, the numerator and the denominator of a given ratio did not necessarily originate from the same blood draw. In routine practice CRP, procalcitonin and albumin are measured on the same biochemistry and immunoassay panel, whereas lactate is obtained by blood gas analysis, so the components of CAR and PAR are drawn from a single routine panel, whereas lactate is measured separately; because sample-level timestamps were not available, the frequency of contemporaneous sampling could not be quantified for any of the three ratios. The ratios are therefore best understood as composite indices of the early inflammatory and physiological state rather than as directly observed biological quantities. They were defined this way deliberately, so that each ratio is derived from the same 24 h window, and from the same convention of taking the most abnormal value, as the SOFA score against which it is compared. A definition based on contemporaneous pairs would not be a more accurate version of the same exposure but a different exposure, measured over a different window from the comparator, and the two analyses would not be directly comparable. The SOFA score was computed from the worst values recorded during the same 24 h window. The pre-specified covariates were the SOFA score, sex and the age-adjusted Charlson Comorbidity Index (CCI) [8]. Together, these constituted the reference (base) prognostic model. This model was specified before any analysis was undertaken and was deliberately parsimonious. It combines the severity of acute organ dysfunction, which is the strongest single determinant of short-term ICU mortality, with the two baseline patient characteristics that are already fixed at the landmark and are not themselves consequences of the acute illness, namely sex and the age-adjusted CCI. Variables that describe the response to critical illness, such as invasive mechanical ventilation, vasopressor support and renal replacement therapy, were deliberately excluded because they are determined after the window in which the ratios are measured and would act as mediators rather than as baseline confounders. Admission diagnosis was also excluded because its categories were numerous and several contained few events, which would have made the reference model unstable. This combination has not previously been validated as a prediction rule in this population; it was specified as a transparent severity baseline against which incremental information could be judged, not as a competing prognostic index. Additional descriptive variables comprised the admission category, individual comorbidities and organ-support exposures (invasive mechanical ventilation, vasopressor support and renal replacement therapy). Sepsis was defined according to the Sepsis-3 criteria as a suspected or documented infection accompanied by an acute increase of 2 points or more in the SOFA score [9]. The septic subgroup comprised admissions classified as sepsis (*n* = 116), and the remaining admissions formed the non-septic subgroup (*n* = 256). Because the criteria were applied retrospectively, infection was taken from the admission diagnosis and the clinical documentation recorded by the treating team, and the change in SOFA score was taken relative to an assumed baseline of zero in patients without documented pre-existing organ dysfunction, as is conventional when a pre-admission score is unavailable. Classification was performed from the clinical record before any outcome analysis was undertaken and was not revised thereafter. Retrospective ascertainment of infection is nonetheless less reliable than prospective adjudication, and some misclassification of the septic subgroup cannot be excluded.

### 2.4. Data Sources and Measurement

Demographic, clinical and laboratory data were retrieved from the hospital information system and the laboratory information system. CRP, procalcitonin and albumin were measured in the hospital’s central clinical laboratory on a Roche cobas 8000 modular analyser series (Roche Diagnostics, Mannheim, Germany), and lactate was measured in whole blood on a Siemens RAPIDLab 1265 blood gas analyser (Siemens Healthineers, Erlangen, Germany); all instruments were operated under routine calibration and internal and external quality control. CRP was determined by a particle-enhanced immunoturbidimetric assay and albumin by a bromocresol green (BCG) dye-binding assay on the clinical chemistry module, procalcitonin by electrochemiluminescence immunoassay (Elecsys BRAHMS PCT, Roche Diagnostics, Mannheim, Germany) on the immunoassay module, and lactate by an amperometric lactate oxidase enzyme electrode. All assays were performed under the laboratory’s routine daily internal quality-control procedures and its participation in an external quality-assessment scheme, and analytical performance was monitored against the manufacturers’ specifications throughout the study period. Analyte-specific coefficients of variation for the study period could not be retrieved retrospectively and are therefore not reported, which is a limitation for the reproducibility of the absolute ratio values and, in particular, of the exploratory cut-offs. The age-adjusted CCI was derived from comorbidities documented before ICU admission and the SOFA score from variables recorded during the first 24 h.

### 2.5. Bias

All consecutive admissions during the study period were screened, and a complete-case approach was applied to the four index laboratory values. A complete-case design does not in itself reduce selection bias and may increase it when the probability of a complete early work-up is related to illness severity or to clinical management; it is described here as the way in which the cohort was assembled, not as a safeguard against bias. The primary outcome (ICU mortality) was objectively documented and was not subject to observer interpretation. Two sources of selection were anticipated and are addressed in the Section 4: the restriction of the cohort to admissions that completed the 24 h landmark, and the requirement for a complete early inflammatory and metabolic work-up. Both were examined empirically rather than left to conjecture, using the limited dataset subsequently obtained for the excluded admissions, as described in Section 2.9.

### 2.6. Study Size

No a priori sample-size calculation was performed; all eligible patients admitted during the one-year study period were included. The 248 deaths observed, relative to the small number of covariates in the base model, provided an events-per-variable ratio well above the conventional minimum of ten.

### 2.7. Quantitative Variables

Continuous variables, which were non-normally distributed, are summarised as the median (interquartile range, IQR). Because the three ratios were right-skewed, they were natural-log-transformed for regression modelling and expressed per one standard deviation (SD) increase. The assumption that each log-transformed ratio was linearly related to the log-odds of mortality was examined with restricted cubic splines. For descriptive classification, marker-specific cut-offs were derived using the Youden index; the corresponding sensitivity, specificity and predictive values represent apparent (in-sample) performance. Because these thresholds were both derived and evaluated in the same cohort they are optimistic, and they are reported as exploratory rather than as clinically applicable cut-offs.

### 2.8. Statistical Analysis

Continuous variables were compared between survivors and non-survivors with the Mann–Whitney U test and categorical variables with the chi-square test. Discrimination for ICU mortality was quantified as the area under the receiver-operating-characteristic curve (AUC) with 95% confidence intervals (CIs), and AUCs were compared using the DeLong test [10]. The incremental value of each ratio was assessed by adding it individually to the base model (SOFA, sex and age-adjusted CCI) in multivariable logistic regression. Nested models were compared by the change in AUC (ΔAUC), the likelihood-ratio test, and the category-free NRI and IDI [11]. CAR and PAR were additionally entered together, and each was reported adjusted for the other. Model calibration was assessed with the Hosmer–Lemeshow test, the Brier score and a bootstrap optimism-corrected calibration slope, and the base + CAR model was internally validated by bootstrapping to obtain an optimism-corrected AUC. All bootstrap procedures, including the confidence intervals reported for the NRI and the IDI, used 1000 resamples. A pre-specified subgroup analysis compared the discrimination of CAR, PAR and LAR between septic and non-septic admissions. Analyses were performed with IBM SPSS Statistics (version 31; IBM Corp., Armonk, NY, USA) and R (version 4.5.1; R Foundation for Statistical Computing, Vienna, Austria). The descriptive comparisons, the areas under the curve and their confidence intervals, the DeLong comparisons of correlated areas, the logistic regression models, the Hosmer–Lemeshow test and the Brier score were carried out independently in both packages, and the resulting estimates agreed to the precision reported here. The category-free NRI and IDI, the bootstrap internal validation and optimism correction, the decision curve analysis and the spline models were performed in R alone, using the pROC, rms, PredictABEL and rmda packages, because these procedures are not implemented in SPSS. To determine whether the prognostic contribution of each ratio reflected the composite rather than its constituents, CRP, procalcitonin, lactate and albumin were each added individually to the base model, and the resulting gains in discrimination were compared with those obtained by adding the corresponding ratio. Collinearity among the base-model covariates and the inflammatory ratios was assessed using variance inflation factors (VIFs). The four primary incremental comparisons, comprising the addition of CAR, PAR, LAR and of CAR and PAR together to the base model, were pre-specified as a family, and their *p*-values are reported both unadjusted and after adjustment for multiplicity by the Bonferroni and the Benjamini–Hochberg methods. The net benefit of adding CAR to the base model was evaluated by decision curve analysis, in which the net benefit of the base and base-plus-CAR models was plotted across a range of threshold probabilities and compared with the treat-all and treat-none strategies. Threshold probabilities in this setting express the predicted risk of ICU death above which a clinician would act on the prediction; because no single action follows from a prognostic estimate of mortality in the way that it does from a diagnostic test, the thresholds are presented as a descriptive range rather than as decision rules, and the analysis is interpreted as a comparison of model behaviour rather than as evidence of clinical benefit. Calibration was displayed graphically for both the base model and the base-plus-CAR model. Two further analyses examined the robustness and context-dependence of the CAR effect: a sensitivity analysis that repeated the incremental-value assessment after excluding patients with a metastatic solid tumour or a haematological malignancy and a formal test of effect modification obtained by adding a sepsis-by-CAR interaction term to the base-plus-CAR model. Departure from linearity in the association between each log-transformed ratio and the log-odds of ICU mortality was assessed by comparing a model containing a linear term with one containing a restricted cubic spline expansion (four knots placed at the 5th, 35th, 65th and 95th percentiles) using the likelihood-ratio test, with three-knot and five-knot specifications examined as a sensitivity check. A two-sided *p*-value < 0.05 was considered statistically significant. Generative artificial intelligence (Claude Opus 5, Anthropic, San Francisco, CA, USA) was used to assist with language refinement and to write the plotting code used to render the figures; it was not used to generate study data, figure content, or statistical results. The authors reviewed and verified all output and take full responsibility for the content of this manuscript.

### 2.9. Analysis of Cohort Selection

Age, sex, admission category, SOFA score, unadjusted Charlson Comorbidity Index, organ-support interventions, ICU length of stay and ICU outcome were retrieved for the 626 admissions that reached the 24 h landmark but lacked one or more index laboratory values and for the 304 admissions that did not reach the landmark. For the 626 admissions, the availability of each of the four analytes was also recorded separately, so that the pattern of missingness could be described. No laboratory values were retrieved for these admissions, since their absence was the reason for exclusion. Because the age-adjusted Charlson Comorbidity Index was not available in the same form for all groups, comparisons were made on the unadjusted index, and the age-adjusted index was reconstructed by adding the conventional age points.

Included and excluded admissions were compared with the Mann–Whitney U test and the chi-square test. To quantify the selection rather than merely describe it, a logistic model of the probability of inclusion was fitted among the 998 admissions that reached the 24 h landmark, with age, sex, SOFA score, unadjusted Charlson Comorbidity Index, admission category, invasive mechanical ventilation, vasopressor support and renal replacement therapy as predictors. Continuous predictors entered the model linearly and admission category as indicator variables with respiratory failure as the reference. For each included patient the fitted probability of inclusion was obtained from this model, and the weight was taken as its reciprocal, so that patients resembling those who were excluded carried greater weight. Weights were stabilised by dividing by their mean, giving a set with a mean of one that preserves the sample size. The weighted sample therefore represents all admissions reaching the landmark rather than only those with a complete work-up. The incremental value of each ratio was then re-estimated under these weights, with robust (sandwich) variance estimation, weighted areas under the curve, and a bootstrap confidence interval for the weighted change in the area obtained from 1000 resamples. Weighted areas under the curve were computed as the weighted proportion of concordant survivor and non-survivor pairs. Calibration was reassessed under the weights using a weighted Hosmer–Lemeshow statistic and a weighted Brier score. The weights themselves were examined before use: their distribution, their coefficient of variation and the effective sample size were recorded, and the balance achieved between the weighted analysis cohort and the full landmark population was assessed with standardised mean differences for every covariate in the model, an absolute value below 0.10 being taken as adequate balance. As a stability check the weights were truncated at the 1st and 99th percentiles and the analysis was repeated. The dependence of the findings on the reference model was examined separately by repeating the incremental analysis against four alternative baselines: the SOFA score alone; the main base model with age entered as a continuous term; the main base model with admission category added; and the main base model with the three organ-support interventions added. The dependence of the findings on the choice of outcome was examined by repeating the incremental analysis with in-hospital mortality in place of ICU mortality. This approach assumes that inclusion is independent of ICU mortality conditional on the measured covariates, an assumption that cannot be verified and is considered in the Section 4.

## 3. Results

### 3.1. Participants and Descriptive Data

During the study period, 372 patients met the eligibility criteria, of whom 124 (33.3%) survived to ICU discharge and 248 (66.7%) died in the ICU; in-hospital mortality was 72.0% (268/372), reflecting a further 20 deaths among ICU survivors before hospital discharge. The cohort carried a high burden of acute and chronic disease: the median age was 70 years (IQR 59–77), the median SOFA score was 9 (IQR 6–12), 22.0% had a metastatic solid tumour and 19.6% a haematological malignancy. Respiratory failure (42.5%) and sepsis (31.2%) were the most common admission categories. Baseline characteristics according to ICU survival are shown in Table 1. Compared with survivors, non-survivors had higher SOFA scores (median 11 versus 7; *p* < 0.001), lower serum albumin (2.5 versus 2.8 g/dL; *p* < 0.001), and higher CRP, procalcitonin and lactate, with correspondingly higher CAR, PAR and LAR (all *p* < 0.001). Non-survivors also more often required invasive mechanical ventilation (89.9% versus 37.1%), vasopressor support (92.3% versus 46.0%) and renal replacement therapy (46.8% versus 27.4%; all *p* < 0.001).

### 3.2. Discrimination of Individual Predictors

As a single predictor, the SOFA score showed the best discrimination for ICU mortality (AUC 0.730, 95% CI 0.677–0.782; Table 2). Among the albumin-anchored ratios, discrimination was moderate and very similar across markers: CAR 0.671 (0.614–0.729), PAR 0.689 (0.630–0.747) and LAR 0.642 (0.585–0.700). The two index ratios were statistically indistinguishable as standalone predictors, with no significant difference between their AUCs (CAR versus PAR, *p* = 0.561), and neither differed significantly from LAR (CAR versus LAR, *p* = 0.415; PAR versus LAR, *p* = 0.184). Serum albumin alone discriminated less well (0.633, 0.573–0.692), as did the age-adjusted Charlson Comorbidity Index (0.552, 0.491–0.614). As standalone predictors, each ratio performed similarly to its inflammatory numerator: CAR (0.671) was only marginally higher than CRP alone (0.656; DeLong *p* = 0.042) and did not differ significantly from albumin alone (0.633; *p* = 0.228), while PAR (0.689) did not differ materially from procalcitonin alone (0.681).

### 3.3. Incremental Value over the Base Model

**Figure 2:**
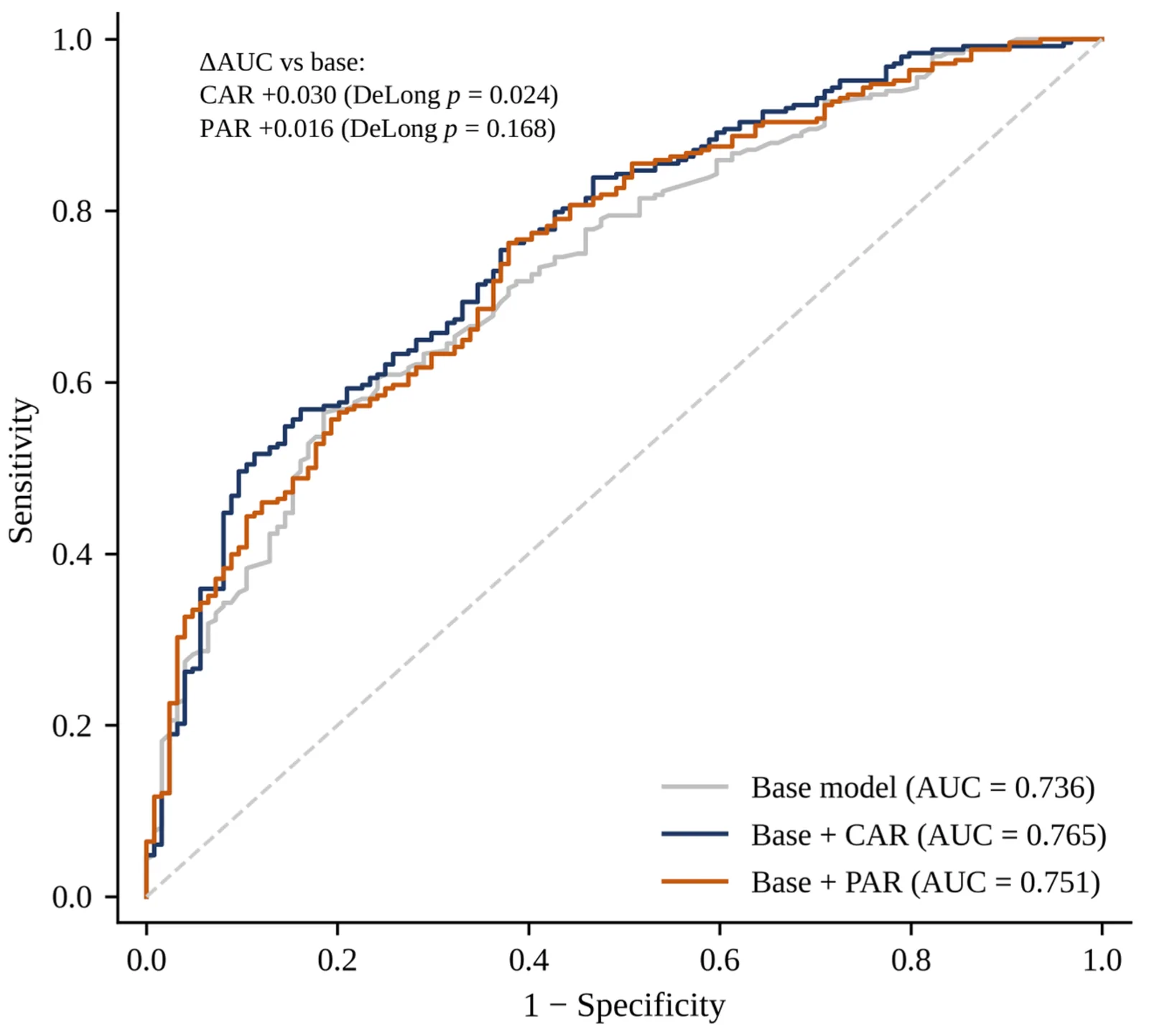
Receiver-operating-characteristic curves for the base model (SOFA, sex and age-adjusted CCI) and for the same model after adding CAR or PAR. Areas under the curve were compared with the DeLong test; the ΔAUC and DeLong *p* are shown on the plot. The base + CAR model was internally validated by bootstrapping (optimism-corrected AUC 0.756). The dashed diagonal line represents the line of no discrimination (AUC = 0.50). AUC, area under the curve; CAR, C-reactive protein-to-albumin ratio; CCI, Charlson Comorbidity Index; PAR, procalcitonin-to-albumin ratio; SOFA, Sequential Organ Failure Assessment.

When each ratio was added individually to the base model (SOFA, sex and age-adjusted CCI), only CAR significantly improved discrimination. Adding CAR raised the AUC to 0.765 (95% CI 0.715–0.815), a gain of +0.030 over the base model (DeLong *p* = 0.024), accompanied by a category-free NRI of +0.39 (95% CI +0.17 to +0.61) and an IDI of +0.042 (+0.013 to +0.091), with good calibration (Hosmer–Lemeshow *p* = 0.69) and a reduced Brier score (0.179 versus 0.189 for the base model). In contrast, adding PAR produced only a non-significant increase in AUC (+0.016; *p* = 0.168), and adding LAR did not improve discrimination either (+0.006; *p* = 0.360). These results are summarised in Table 3, and the corresponding ROC curves are shown in Figure 2.

To establish whether the incremental value of CAR reflected the composite ratio rather than either component, CRP, procalcitonin and albumin were each added individually to the base model. Only CAR and CRP produced a significant improvement in discrimination (base + CAR, ΔAUC +0.030, *p* = 0.024; base + CRP, ΔAUC +0.025, *p* = 0.033), whereas adding albumin alone (ΔAUC +0.018, *p* = 0.161) or procalcitonin alone (ΔAUC +0.013, *p* = 0.211) did not; CAR provided the largest gain among the candidates tested. Collinearity was negligible: the correlation between log-transformed CAR and PAR was moderate (r = 0.53), as was that between log CRP and log procalcitonin (r = 0.48), and all variance inflation factors in the base + CAR + PAR model were below 1.7. The complete specification of every logistic model reported here, including intercepts, coefficients and standard errors, is given in Appendix A.

**Figure 3:**
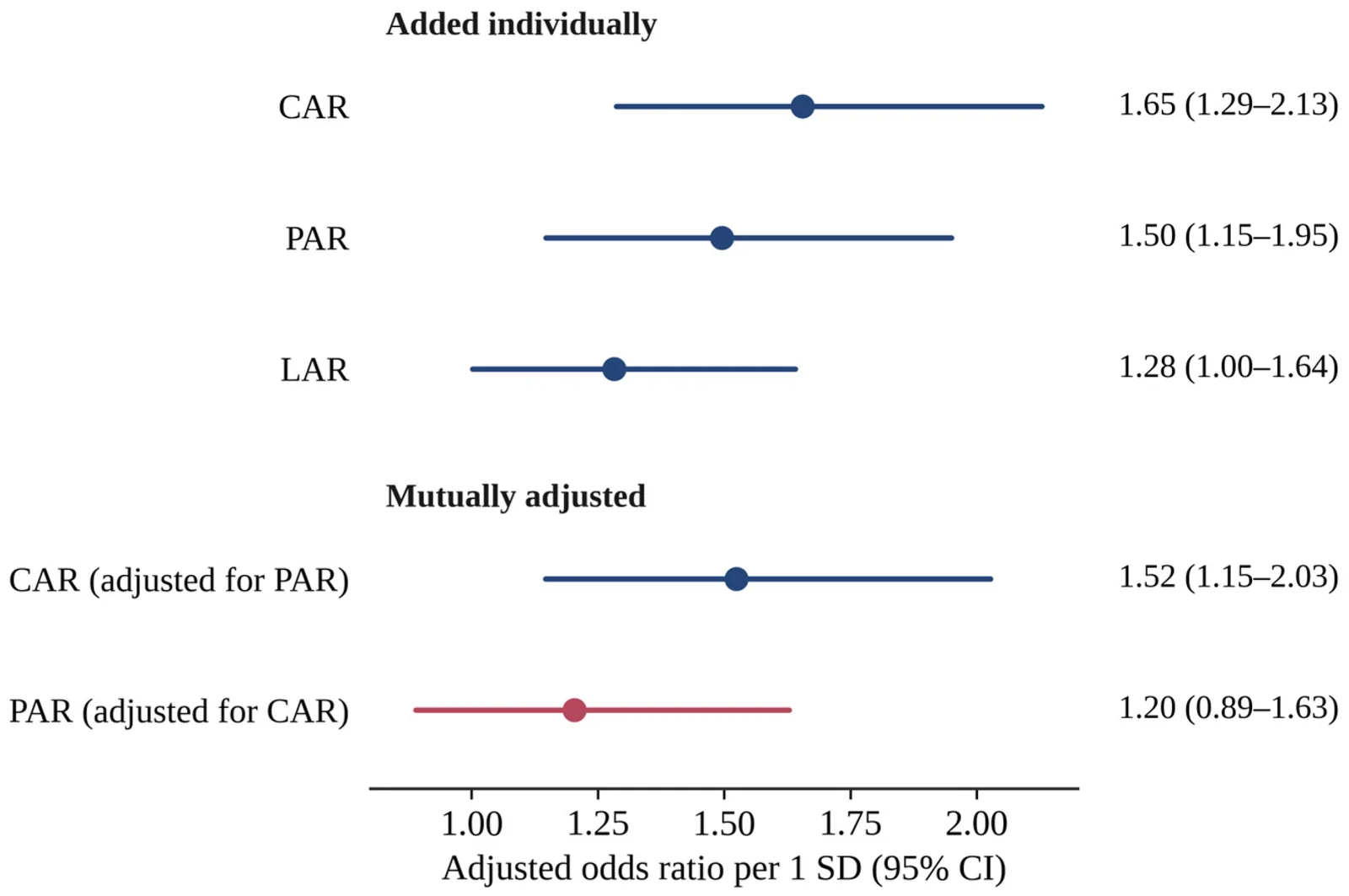
Adjusted odds ratios for ICU mortality, expressed per 1 SD of the log-transformed ratio. Each ratio was added individually to the base model (SOFA, sex and age-adjusted CCI; upper section), and CAR and PAR were also entered together (lower section). Points are odds ratios and horizontal bars are 95% CIs; estimates whose CI excludes 1 are shown in blue and the non-significant estimate in red. CAR, C-reactive protein-to-albumin ratio; CCI, Charlson Comorbidity Index; CI, confidence interval; LAR, lactate-to-albumin ratio; PAR, procalcitonin-to-albumin ratio; SD, standard deviation; SOFA, Sequential Organ Failure Assessment.

The independence of CAR was confirmed when both inflammatory ratios were modelled together. With CAR and PAR entered simultaneously, CAR remained independently associated with ICU mortality (adjusted odds ratio 1.52 per 1 SD, 95% CI 1.15–2.03), whereas PAR did not. The combined base + CAR + PAR model discriminated no better than base + CAR (AUC 0.768) and was less well calibrated (Hosmer–Lemeshow *p* = 0.02), indicating that PAR did not provide statistically significant independent information once CAR was accounted for. The adjusted odds ratios for the three ratios are displayed in Figure 3.

**Figure 4:**
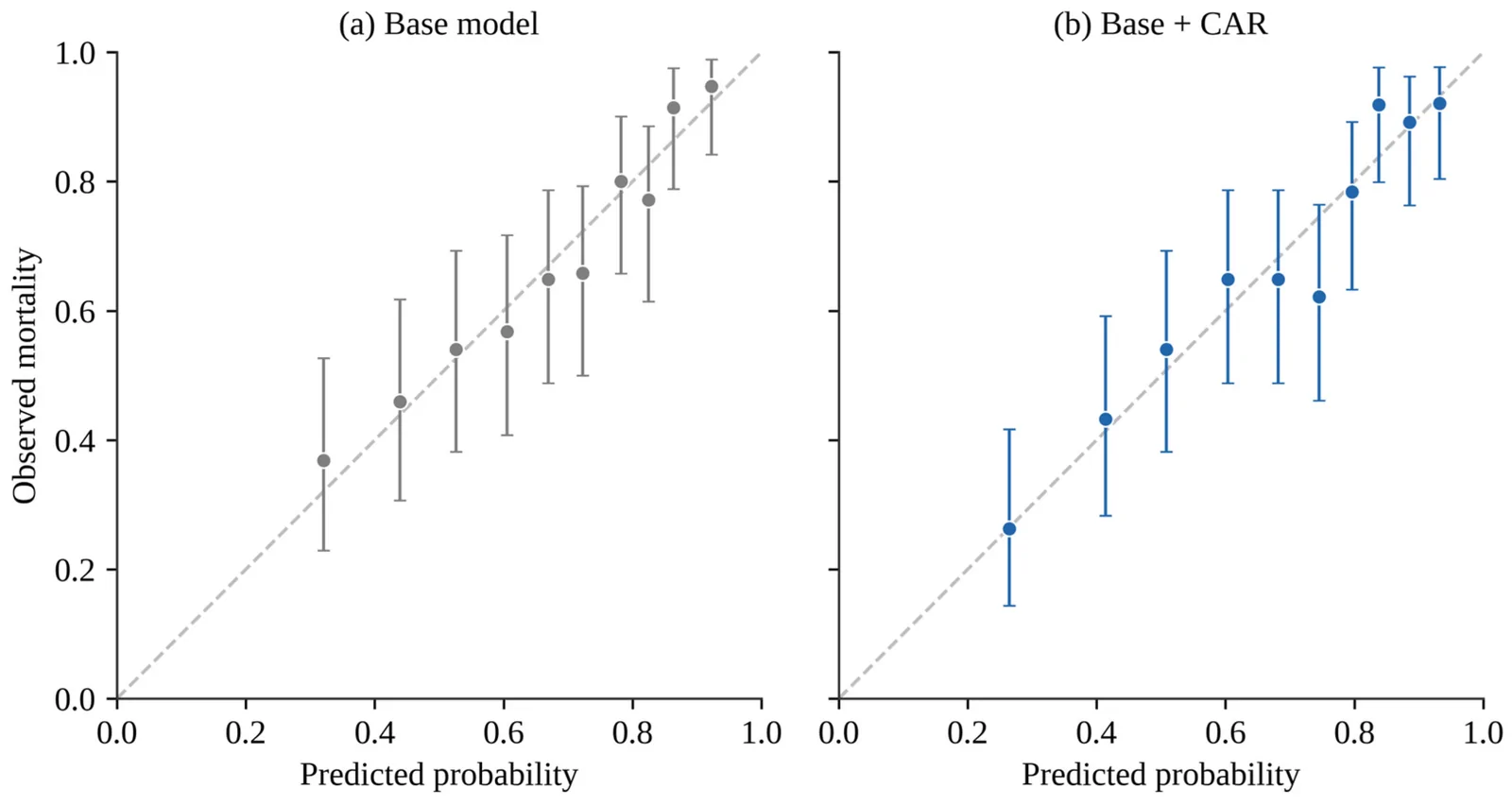
Calibration of the base model (**a**) and the base + CAR model (**b**); base model = SOFA, sex and age-adjusted CCI. Observed mortality is plotted against the mean predicted probability across deciles of predicted risk; the dashed diagonal denotes perfect calibration and the error bars show 95% CIs. Grey markers denote the base model and blue markers the base + CAR model; the colours distinguish the two panels and carry no additional meaning. Both models were well calibrated (Hosmer–Lemeshow *p* = 0.91 and *p* = 0.69, respectively); the base + CAR model had a bootstrap optimism-corrected calibration slope of 0.95. CAR, C-reactive protein-to-albumin ratio; CCI, Charlson Comorbidity Index; CI, confidence interval; SOFA, Sequential Organ Failure Assessment.

The base + CAR model was internally validated by bootstrapping, which yielded an optimism-corrected AUC of 0.756 and a calibration slope of 0.95, indicating minimal overfitting. Observed versus predicted mortality across deciles of predicted risk is shown in Figure 4.

### 3.4. Net Benefit and Calibration

**Figure 5:**
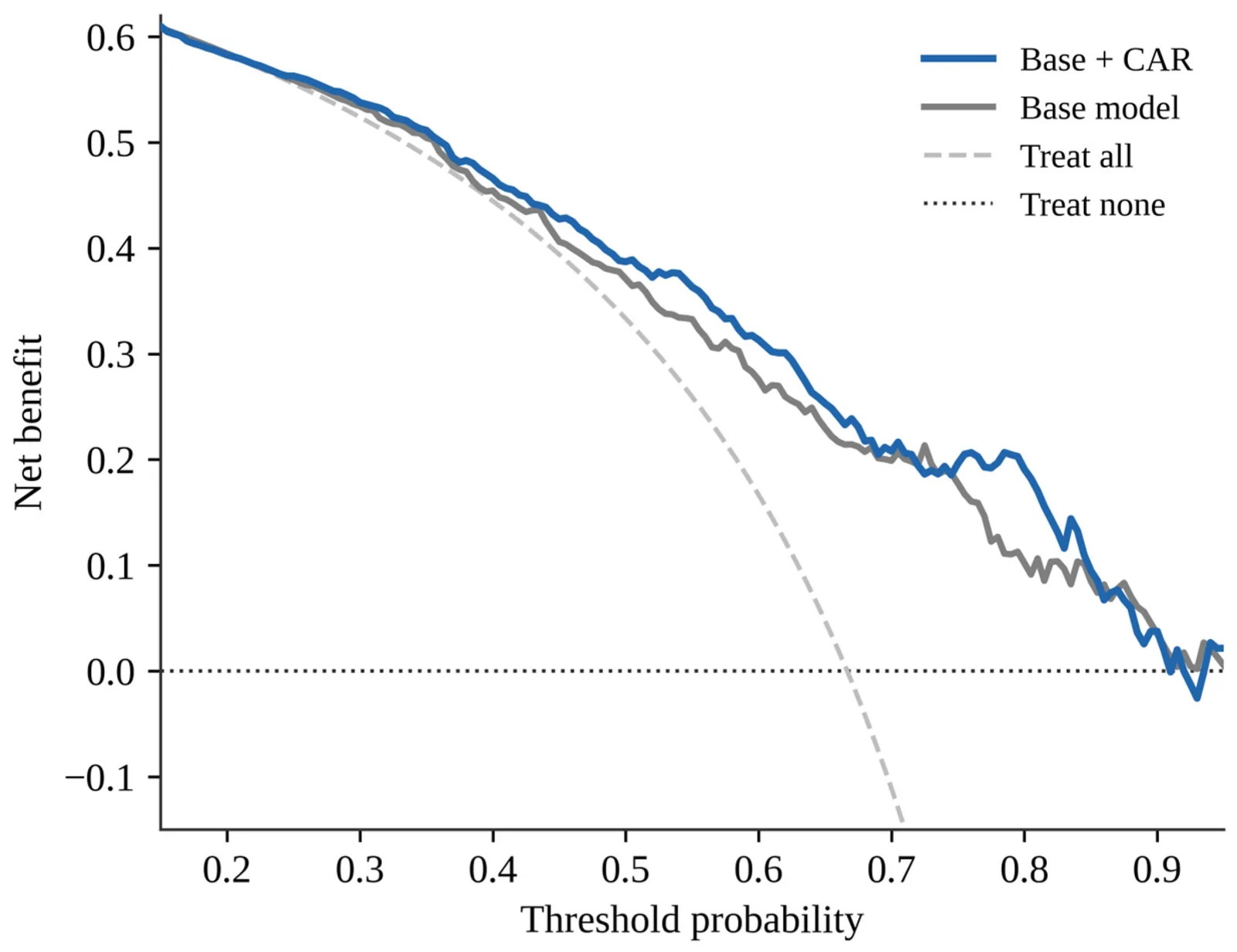
Decision curve analysis comparing the net benefit of the base model (SOFA, sex and age-adjusted CCI) and the base + CAR model across threshold probabilities for ICU mortality. The treat-all and treat-none strategies are shown for reference. Adding CAR increased the net benefit across the clinically relevant threshold range. CAR, C-reactive protein-to-albumin ratio; CCI, Charlson Comorbidity Index; SOFA, Sequential Organ Failure Assessment.

On decision curve analysis, adding CAR to the base model increased the net benefit across the clinically relevant range of threshold probabilities (approximately 0.30 to 0.90), and both models exceeded the treat-all and treat-none strategies throughout this range (Figure 5). Calibration was good for both the base model (Hosmer–Lemeshow *p* = 0.91) and the base + CAR model (*p* = 0.69), as displayed in Figure 4. Decision curve analysis describes how a model would behave if used at a given decision threshold and cannot by itself demonstrate that measuring CAR changes management or improves outcome. The range over which the advantage was seen, roughly 0.30 to 0.90, lies around and above the observed ICU mortality of 66.7%, so it describes the behaviour of the models across risks that are common in this cohort rather than identifying a threshold at which a specific action would be taken. No clinical action follows automatically from a predicted probability of ICU death, and the thresholds should therefore be read descriptively.

### 3.5. Exploratory Cut-Offs and Classification Performance

Youden-derived cut-offs for the three ratios are presented in Table 4; these reflect apparent (in-sample) performance and are exploratory. These thresholds were derived and evaluated in the same cohort, so they are optimistic and are not proposed as clinically applicable cut-offs. A CAR threshold of 45.9 separated patients at higher and lower risk of ICU death (mortality 79% above versus 54% below the threshold), with a sensitivity of 0.60 and a specificity of 0.68. The PAR (0.48) and LAR (0.93) thresholds performed similarly, with sensitivities of 0.64 and 0.51 and specificities of 0.68 and 0.78, respectively. Because cohort mortality was high (66.7%), positive predictive values were high (0.79–0.82) and negative predictive values modest (0.44–0.48) for all three markers.

### 3.6. Septic Versus Non-Septic Subgroups

**Figure 6:**
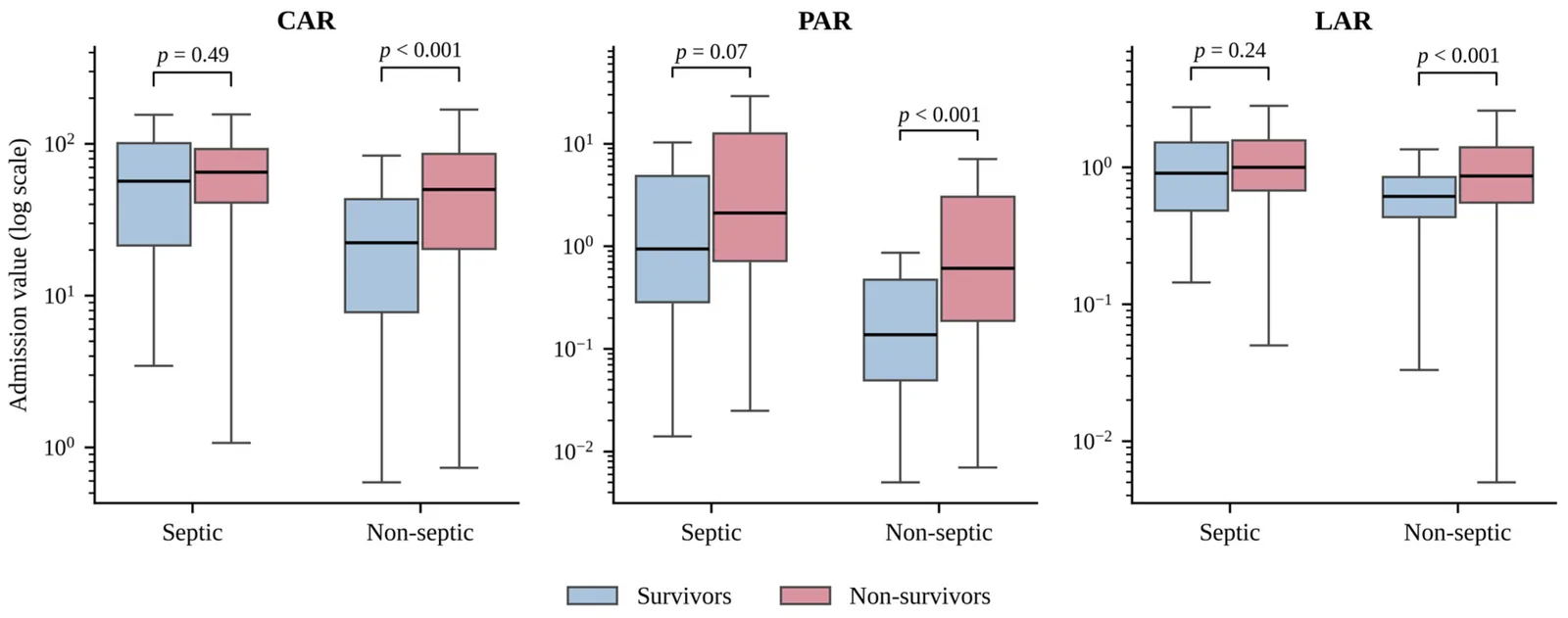
First 24 h CAR, PAR and LAR by ICU survival in septic and non-septic patients (log scale). Boxes show the median and IQR and the whiskers extend to 1.5 times the IQR; within each subgroup, survivors were compared with non-survivors using the Mann–Whitney U test, with *p* shown above each pair. CAR, C-reactive protein-to-albumin ratio; ICU, intensive care unit; IQR, interquartile range; LAR, lactate-to-albumin ratio; PAR, procalcitonin-to-albumin ratio.

The discrimination of all three ratios differed between septic and non-septic admissions (Table 5). In the 116 septic patients (mortality 74%), none of the ratios discriminated well (CAR AUC 0.543, PAR 0.610, LAR 0.572), and admission marker levels did not differ significantly between survivors and non-survivors. In the 256 non-septic patients (mortality 63%), all three ratios discriminated meaningfully (CAR 0.698, PAR 0.702, LAR 0.652), and survivors had significantly lower CAR, PAR and LAR than non-survivors (all *p* < 0.001). Although absolute marker levels were higher in septic than in non-septic admissions (for example, median CAR 64.3 versus 36.0; both *p* ≤ 0.001), their prognostic value was paradoxically lower in the septic group. The distribution of the three ratios by survival within each subgroup is shown in Figure 6.

### 3.7. Sensitivity and Interaction Analyses

In a sensitivity analysis restricted to the 219 patients without a metastatic solid tumour or haematological malignancy (mortality 58.0%), the direction and magnitude of the CAR effect were preserved (adjusted odds ratio 1.69 per SD, 95% CI 1.23 to 2.33; base AUC 0.752 rising to 0.779 with CAR), although the gain in discrimination no longer reached significance in this smaller subset (ΔAUC +0.027, DeLong *p* = 0.090). A formal sepsis-by-CAR interaction term was not significant (*p* = 0.333), indicating that the divergent subgroup performance should be regarded as hypothesis-generating rather than as statistically confirmed effect modification. There was no evidence of departure from linearity for CAR (likelihood-ratio *p* = 0.51) or for PAR (*p* = 0.43) when each log-transformed ratio was modelled with restricted cubic splines, and this was unchanged with three-knot and five-knot specifications. For LAR there was some evidence of non-linearity (*p* = 0.03 with four knots, *p* = 0.01 with three knots and *p* = 0.07 with five knots), but modelling LAR flexibly still did not significantly improve discrimination over the base model (AUC 0.752, ΔAUC +0.017, DeLong *p* = 0.15), so the interpretation of LAR is unchanged. When in-hospital mortality (268 deaths, 72.0%) replaced ICU mortality as the outcome, the incremental contribution of CAR was preserved and somewhat larger (base AUC 0.730 rising to 0.774; ΔAUC +0.044, DeLong *p* = 0.007; adjusted odds ratio 1.84 per SD, 95% CI 1.41 to 2.39). For this outcome, however, PAR also improved discrimination (+0.033, *p* = 0.036) and retained independence when entered together with CAR (adjusted odds ratio 1.42 per SD, 95% CI 1.03 to 1.97, *p* = 0.034), which it did not for ICU mortality. The contrast between the two ratios is therefore specific to the outcome studied and should not be generalised to mortality prediction in general. After adjustment of the four primary incremental comparisons for multiplicity, the increment attributable to CAR retained significance under the Benjamini–Hochberg procedure (adjusted *p* = 0.049) but not under the Bonferroni correction (adjusted *p* = 0.098); the comparisons for PAR and LAR remained non-significant under both.

### 3.8. Cohort Selection and Selection-Weighted Sensitivity Analysis

Of the 304 admissions that did not reach the 24 h landmark, 158 (52.0%) died in the ICU and 146 (48.0%) were discharged to a ward. Among the 626 admissions excluded for an incomplete work-up, no patient had all four analytes available: 478 (76.4%) lacked one analyte, 142 (22.7%) lacked two and six (1.0%) lacked three or four. Procalcitonin was the analyte most often missing (376 admissions, 60.1%), followed by albumin (250, 39.9%), CRP (125, 20.0%) and lactate (31, 5.0%). Demographic or outcome data were missing for only four admissions. A sensitivity analysis adding excluded admissions with a complete biomarker panel was therefore not possible, because no such admission existed.

Table 6 compares the analysis cohort with both excluded groups. Included and excluded admissions were of the same age (median 70 years in each group) and had the same comorbidity burden (unadjusted Charlson Comorbidity Index 4 in each group), but the analysis cohort was more severely ill: the median SOFA score was 9 versus 8 (*p* < 0.001), vasopressor support had been given to 76.9% versus 61.2% (*p* < 0.001), invasive mechanical ventilation to 72.3% versus 64.4% (*p* = 0.012) and renal replacement therapy to 40.3% versus 31.8% (*p* = 0.008). Sepsis was the admission category in 31.2% of included and 55.9% of excluded admissions (*p* < 0.001). ICU mortality did not differ significantly between the two groups (66.7% versus 62.0%, *p* = 0.155).

The logistic model of inclusion discriminated moderately (AUC 0.737), confirming that selection was not random. Inclusion was more likely with increasing organ dysfunction (odds ratio 1.09 per SOFA point, 95% CI 1.05 to 1.14) and with vasopressor support (2.50, 1.69 to 3.70) and was less likely for admissions with sepsis than for those with respiratory failure (0.28, 0.20 to 0.40) and for men (0.71, 0.53 to 0.95). Age, comorbidity, mechanical ventilation and renal replacement therapy were not independently associated with inclusion. The full model is given in Appendix A, Table A2. The resulting weights were well behaved: they ranged from 0.39 to 6.12 with a median of 0.73 and a coefficient of variation of 0.75, no weight exceeded 6.2, and the effective sample size was 239 of 372 patients (64.2%). Before weighting, the analysis cohort differed from the full landmark population most in sepsis (standardised mean difference −0.32), vasopressor support (+0.22) and SOFA score (+0.22). After weighting, every covariate in the model was balanced to within 0.12 and all but two to within 0.10 (sepsis +0.10, mechanical ventilation −0.11), indicating that the weights removed the measured component of the selection. The full balance table is given in Appendix A, Table A3.

When the analysis cohort was reweighted to represent all 998 admissions reaching the landmark, the weighted ICU mortality fell from 66.7% to 59.1%. CAR remained independently associated with ICU mortality after weighting (adjusted odds ratio 1.47 per SD, 95% CI 1.13 to 1.92, *p* = 0.004), but its incremental discrimination was attenuated: the weighted AUC rose from 0.756 for the base model to 0.774 with CAR, a gain of +0.018 (bootstrap 95% CI +0.000 to +0.050) compared with +0.030 in the unweighted analysis. Truncating the weights at the 1st and 99th percentiles gave essentially the same result (+0.019). Calibration was preserved under weighting: the weighted Hosmer–Lemeshow statistic gave *p* = 0.26 for the base model and *p* = 0.86 for the base + CAR model, and the weighted Brier score was 0.193 and 0.188 respectively. LAR again added nothing under weighting (+0.002). The dependence of the finding on the reference model was examined separately. Against the SOFA score alone, CAR raised the area under the curve from 0.730 to 0.763 (ΔAUC +0.033, DeLong *p* = 0.020), and the result was unchanged when age was added to the main base model as a continuous term (+0.029, *p* = 0.030). Against richer baselines the increment was smaller and no longer reached significance: adding the admission category to the base model raised its area to 0.760, after which CAR contributed +0.018 (*p* = 0.105), and adding the three organ-support interventions raised it to 0.841, after which CAR contributed +0.018 (*p* = 0.079). In all four models the adjusted odds ratio for CAR remained significant and of similar magnitude (1.53 to 1.68 per SD).

## 4. Discussion

In this retrospective cohort of 372 high-acuity medical ICU patients, three albumin-anchored ratios from the first 24 h were compared as predictors of ICU mortality. As standalone markers, CAR, PAR and LAR provided moderate and statistically indistinguishable discrimination, and all were inferior to the SOFA score. The key distinction emerged in discrimination and in joint modelling: only CAR significantly improved the area under the curve beyond a model comprising SOFA, sex and the age-adjusted Charlson Comorbidity Index, and only CAR remained independently associated with mortality when the two ratios were entered together. PAR produced positive reclassification metrics whose confidence intervals excluded zero, but it did not significantly improve discrimination and lost independence alongside CAR. Finally, all three ratios discriminated in non-septic admissions, but their discriminative ability could not be demonstrated in septic admissions, despite higher absolute marker concentrations in the latter.

These findings should be read against the literature that has largely equated prognostic relevance with statistical association or crude discrimination. CAR is the best studied of the three ratios and has repeatedly been reported as an independent predictor of mortality across medical, postoperative and septic ICU populations [4,12,13]. A pooled prognostic association has likewise been confirmed in meta-analysis [14]. Yet where CAR has been compared directly with established predictors, its discrimination has been modest and at times no greater than that of serum albumin or generic severity indices alone [15]. An increase in the area under the curve is, moreover, an insensitive way to judge a new marker when strong predictors are already present, which is precisely why reclassification and calibration metrics are recommended [11,16]. By quantifying the change in AUC together with the category-free NRI, the IDI, formal calibration and bootstrap internal validation, the present analysis moves beyond association to ask whether these ratios add value to a severity baseline; the results identify CAR, but not PAR or LAR, as a candidate prognostic adjunct that now requires external validation. CAR provided the largest gain among the candidates tested. CRP alone also significantly improved discrimination, whereas albumin and procalcitonin alone did not, so the advantage of the composite over its numerator is one of degree rather than of kind. Decision curve analysis indicated a modestly higher net benefit across the plausible range of decision thresholds, although such an analysis describes the behaviour of a model at a given threshold and cannot establish that measuring CAR would change management or improve outcomes.

The dissociation between CAR and PAR is consistent with, and extends, recent evidence. In a tertiary cardiac ICU that evaluated the same three ratios simultaneously, LAR and CAR each remained independently associated with ICU mortality after adjustment, whereas PAR did not [5]. PAR has shown prognostic value in selected infection-defined cohorts, but such studies have generally examined it in isolation or only in combination with a severity score, rather than in direct competition with a CRP-based ratio within the same model [6,17]. When that competition is performed, as here, PAR adds little once CAR is known, and the combined CAR-plus-PAR model was less well calibrated than the parsimonious base-plus-CAR model, providing no evidence that adding PAR to CAR improves the prediction of ICU mortality in this cohort. We make no recommendation about whether procalcitonin should be measured, and none of this bears on its established diagnostic and antimicrobial-stewardship roles, which lie outside the scope of the present analysis.

Several mechanisms may explain why CAR carries independent prognostic information while PAR does not. Procalcitonin is primarily a marker of bacterial infection with limited prognostic value from a single admission value in established sepsis, where mortality tracks the failure of procalcitonin to fall over time rather than its initial concentration [18,19]. Where infection is common, early PAR may already be elevated in survivors and non-survivors alike, compressing its dynamic range. C-reactive protein, by contrast, is a more graded acute-phase reactant reflecting the systemic inflammatory response [20,21], while albumin adds complementary information on capillary leak and physiological reserve, with hypoalbuminaemia being among the most robust laboratory predictors of death in acute illness [22,23]. Dividing a graded inflammatory numerator by this reserve denominator plausibly yields a more discriminating composite than placing a near-saturated infection marker over the same denominator.

The pattern seen across septic and non-septic admissions is reported for completeness rather than as a subgroup-specific finding and is the least secure observation in this manuscript. Although CAR, PAR and LAR were all higher in septic patients, none separated survivors from non-survivors within the septic subgroup, whereas all three discriminated in non-septic admissions. The septic subgroup was small (*n* = 116), however, and the confidence intervals around its subgroup AUCs were wide, so these data indicate that prognostic value could not be demonstrated in sepsis rather than that it is absent. Consistent with this, a formal sepsis-by-CAR interaction term was not statistically significant, so the apparent subgroup divergence should be interpreted as hypothesis-generating rather than as confirmed effect modification. A further consideration, which the analysis of cohort selection now makes explicit, is that septic admissions were substantially less likely than others to have a complete early panel and therefore to enter the cohort at all. The septic subgroup analysed here is consequently a selected fraction of the septic admissions to this unit, and the failure to demonstrate discrimination within it may reflect that selection as well as any biological ceiling effect. This subgroup finding is therefore the least secure result in the manuscript and requires replication in a cohort in which septic and non-septic admissions are sampled equally. An analogous phenomenon has been described for other biomarkers, which can be uniformly and near-maximally elevated in septic shock and thereby lose the ability to differentiate outcomes, a prognostic ceiling effect, while retaining discrimination in non-septic critical illness [18,24]. In an already maximally inflamed septic population, an early inflammatory ratio may simply have too little residual dynamic range to stratify risk, whereas in non-septic critical illness the same ratio still captures meaningful between-patient variation in the host response. This interpretation should be regarded as hypothesis-generating and requires confirmation in larger, dedicated cohorts.

The high ICU mortality observed here (66.7%) reflects the case-mix of a tertiary medical ICU serving a referral population with a heavy oncological and haematological burden, high illness severity and extensive organ support. Mortality of this magnitude is well precedented in comparable populations: tertiary cohorts of critically ill patients with haematological malignancy report ICU mortality ranging from approximately 45% to over 80% [25,26]. Short-term ICU survival in such patients is, moreover, governed chiefly by the burden of acute organ failure, namely mechanical ventilation, vasopressor support and renal replacement together with the SOFA score, rather than by the underlying malignancy label [27]. The acuity of the present cohort (median SOFA 9, with 72% ventilated, 77% on vasopressor support and 40% receiving renal replacement) is therefore consistent with the observed mortality and defines the population to which these findings apply.

### 4.1. Strengths and Limitations

This study has several strengths, including a pre-specified reference model, the simultaneous head-to-head evaluation of three ratios, and an analysis plan emphasising incremental value, calibration and internal validation rather than association alone. Some considerations should nonetheless be kept in mind when interpreting the results. The study was conducted at a single centre and was retrospective, so external, multicentre validation is the natural next step before clinical adoption, as for any newly described prognostic marker [28]. Selection is the principal limitation of this study, and it has two components. First, the cohort was restricted to admissions that completed the 24 h landmark, and 304 of the 1352 screened admissions did not. This restriction follows from the measurement definitions rather than from the outcome, since both the SOFA score and each ratio are defined on the worst component values observed across a full 24 h period; including shorter stays would have meant comparing exposures measured over unequal and truncated windows. The consequence, which we state explicitly, is that these estimates apply to patients who are still in the ICU 24 h after admission, which is also the point at which the risk assessment considered here would be made, and not to patients who die or are discharged within the first day. Admissions that did not reach the landmark were more often discharged alive and less often received organ support than the analysis cohort, and 52.0% of them died in the ICU; their exclusion therefore removes part of the risk spectrum, although the direction of the resulting effect on discrimination cannot be inferred from these figures alone. Second, 626 admissions were excluded because one or more of the variables required to compute the SOFA score and all four ratios were unavailable within the first 24 h, which may have selected for more intensively monitored and potentially more severely ill patients, so the resulting cohort may under-represent the heterogeneity of the source population. This selection has now been characterised rather than assumed. The analysis cohort was of the same age and carried the same comorbidity burden as the excluded admissions but was more severely ill and more often supported with vasopressors, mechanical ventilation and renal replacement therapy, while ICU mortality did not differ significantly between the groups. The inclusion model confirmed that selection was driven by illness severity and by admission category rather than by demographic factors, and that admissions with sepsis were markedly less likely to have a complete work-up. The complete-case design does not by itself remedy this. Applying the same objective criteria uniformly to every consecutive admission makes the selection transparent and independent of the outcome, but it does not make the analysed cohort representative of the source population. When the cohort was reweighted to represent all admissions reaching the landmark, CAR remained independently associated with ICU mortality but its incremental discrimination fell from +0.030 to +0.018, with a bootstrap confidence interval extending to approximately zero at the reported precision. The principal finding is therefore directionally robust to the measured component of the selection, but its magnitude is smaller in the wider population than in the analysis cohort, and it should be interpreted accordingly. Weighting can only address selection that is captured by the recorded variables; it cannot exclude selection acting through characteristics that were not measured, and residual selection bias therefore remains possible. External validation in prospectively assembled, less selected cohorts is required before these findings are generalised. The exceptionally high mortality and illness severity of this cohort further mean that the findings should not be generalised to lower-acuity, mixed or surgical ICU populations. The improvement in discrimination obtained by adding CAR, although statistically significant, was modest (ΔAUC 0.030). Reclassification metrics such as the NRI and IDI have recognised limitations and can overstate the practical impact of a new marker, so we interpreted them alongside this modest ΔAUC, formal calibration and bootstrap optimism correction [29]. The present data illustrate the point: PAR produced positive NRI and IDI estimates whose confidence intervals excluded zero, yet its ΔAUC was not significant and it lost independence once CAR was entered. Significant reclassification metrics were therefore not treated as independent evidence of clinical benefit, and the primary emphasis was placed on discrimination, calibration and the need for external validation. The four primary incremental comparisons were also examined as a family: the increment attributable to CAR survived Benjamini–Hochberg adjustment but not the more conservative Bonferroni correction, which is a further reason to regard it as small rather than firmly established. The pairwise discrimination comparisons and subgroup analyses were not adjusted for multiple testing and should therefore be regarded as exploratory. The increment attributable to CAR also depends on how rich the reference model is. It was significant against the SOFA score alone and against the pre-specified base model, but fell below significance when admission category or the organ-support interventions were added, even though the adjusted odds ratio for CAR was of similar size in every model. Part of the information CAR carries is therefore shared with the admission diagnosis and with the intensity of early organ support, and the increment reported here should be understood as the value added to a deliberately parsimonious severity baseline rather than to the fullest model a clinician could construct. In keeping with the SOFA scoring window, each ratio was derived from the most abnormal component value within the first 24 h; because CRP and procalcitonin may peak later, this approach could underestimate the full inflammatory response and, potentially, the prognostic value of the ratios. For the same reason the ratios are described throughout as first 24 h rather than admission measurements: they may incorporate clinical deterioration or the effects of treatment given after ICU admission, and the numerator and denominator of a given ratio were not necessarily obtained from the same blood draw. We did not have access to sample-level timestamps for the analysis cohort and therefore cannot report how often the components of a given ratio came from the same draw, nor recompute the ratios from contemporaneous pairs. This is a genuine limitation of the exposure definition, and the ratios should be read as constructed early-phase indices rather than as biological ratios measured at a point in time. Two observations bound its likely consequences. First, the components of CAR and PAR are analysed on the same routine biochemistry and immunoassay panel, whereas lactate is obtained separately by blood gas analysis; the frequency with which the components of a given ratio were in fact drawn together could not be verified. Second, the component analysis reported in Section 3.3 shows that the incremental information in CAR is carried mainly by its numerator, CRP, which retained significant incremental discrimination when entered alone, whereas albumin alone did not; the composite construction is therefore not generating a signal that its components lack. A prospective study using paired, time-stamped measurements would nonetheless settle the question and is the appropriate next step. Albumin was measured with a bromocresol green dye-binding assay, which is known to give slightly higher values than bromocresol purple or immunochemical methods, particularly in the presence of the acute-phase globulins that characterise critical illness. Because albumin is the denominator of all three ratios, the absolute values reported here, and in particular the exploratory cut-offs, are dependent on the albumin method used, and studies seeking to validate these ratios externally should report their own albumin assay. Detailed treatment exposures such as antibiotic timing and fluid balance were not captured and could not be modelled, although the major organ-support interventions were recorded and described. Finally, the ratios were derived from a single early measurement, whereas the dynamic behaviour of these markers is known to carry additional prognostic information [18,19]. Serial or trajectory-based ratios may therefore prove more informative and represent a promising direction for future work. Finally, ICU mortality was the primary outcome and in-hospital mortality was examined only as a sensitivity outcome; whether either ratio carries comparable incremental value for other clinically relevant endpoints remains to be established. That sensitivity analysis preserved and slightly increased the contribution of CAR, but PAR also retained independent value within it, so the contrast between the two ratios is specific to ICU mortality and should not be presented as a general property of the markers.

### 4.2. Generalisability and Scope of Inference

The population to which these results apply is narrower than the population screened. Of 1352 consecutive admissions, 372 contributed to the analysis; the remainder either did not reach the 24 h landmark or did not have a complete early inflammatory and metabolic work-up. The findings therefore describe adults who are alive and in a medical ICU at 24 h and in whom CRP, procalcitonin, albumin and lactate have all been measured within that window in a single high-acuity tertiary unit with an ICU mortality of 66.7%. They should not be extrapolated to unselected intensive care populations, to units with a lower acuity or a different case mix, or to patients in whom this panel is not obtained as part of routine care. The analysed cohort differs from the wider landmark population in a specific and now measurable way: it is more severely ill and contains proportionally fewer septic admissions, because a complete early panel was less often obtained in sepsis. Reweighting to that wider population preserved the direction of the CAR effect but reduced its incremental discrimination by roughly a third, which is the best available estimate of how these results would transport to a less selected medical ICU population reaching the same landmark. For these reasons the present results are offered as hypothesis-generating rather than as a validated prognostic tool, and prospective evaluation in cohorts assembled without these eligibility requirements is the necessary next step. The requirement for a complete panel is itself a feature of local practice rather than of the patients, so the transportability of these findings will depend in part on how consistently procalcitonin and albumin are requested in the receiving unit.

Taken together, these results suggest that the first 24 h CAR, an inexpensive ratio derived from tests performed routinely during the first day of ICU care, may complement rather than replace the SOFA score for risk stratification at the 24 h landmark, with a descriptive pattern of better discrimination in non-septic admissions, although no statistically significant effect modification by sepsis was demonstrated. For ICU mortality, they also indicate that little is gained by adding PAR once CAR is available. The improvement afforded by CAR was modest in absolute terms, was attenuated by roughly a third when the cohort was reweighted to the wider landmark population, and did not persist as a statistically significant increment against richer reference models. It should therefore not be described as clinical utility. What the data support is that CAR is a candidate prognostic adjunct whose incremental contribution is small and conditional on the baseline against which it is judged, and whether it is sufficient to alter bedside decisions or patient outcomes is unknown. Whether incorporating the first 24 h CAR into bedside risk assessment improves clinical decision-making will require prospective, externally validated evaluation.

## 5. Conclusions

Among three albumin-anchored ratios from the first 24 h evaluated in a high-acuity medical ICU, CAR, PAR and LAR were comparable and only moderate as standalone predictors of ICU mortality, yet only CAR significantly improved discrimination beyond a parsimonious severity model based on SOFA, sex and the age-adjusted Charlson Comorbidity Index, and only CAR remained independently informative when modelled together with PAR. That increment was small and survived correction for multiplicity only under the less conservative of the two procedures applied. It was attenuated after weighting for cohort selection and did not remain significant against richer reference models that included admission diagnosis or early organ support. PAR offered no independent value once CAR was accounted for when the outcome was ICU mortality, although it did when the outcome was in-hospital mortality, so this contrast is specific to the endpoint studied. No claim of subgroup specificity is made: discrimination could not be demonstrated within the smaller septic subgroup, interaction testing did not support differential performance, and septic admissions were under-represented in the analysis cohort. The first 24 h CAR may therefore represent a simple, inexpensive candidate prognostic adjunct to complement, rather than replace, the SOFA score at the 24 h landmark; it should not be adopted clinically before external validation. These conclusions apply to patients who remain in the ICU at 24 h and in whom a complete early inflammatory and metabolic work-up is available, and they are hypothesis-generating rather than confirmatory. These single-centre findings require prospective, multicentre external validation, and future work should establish whether serial or trajectory-based ratios outperform a single early measurement.

## Figures and Tables

**Table 1:** Characteristics of the study cohort in the first 24 h of ICU care, according to ICU survival.

Characteristic	All (*n* = 372)	Survivors (*n* = 124)	Non-Survivors (*n* = 248)	*p*
*General characteristics*				
Age, years	70 (59–77)	70 (58–77)	69 (59–78)	0.934
Male sex	200 (53.8)	68 (54.8)	132 (53.2)	0.854
*Admission category, n (%)*				
Respiratory failure	158 (42.5)	46 (37.1)	112 (45.2)	<0.001
Sepsis	116 (31.2)	30 (24.2)	86 (34.7)	
Gastrointestinal/hepatic	36 (9.7)	21 (16.9)	15 (6.0)	
Cardiovascular	18 (4.8)	3 (2.4)	15 (6.0)	
Postoperative/trauma	10 (2.7)	6 (4.8)	4 (1.6)	
Neurologic	8 (2.2)	4 (3.2)	4 (1.6)	
Metabolic/endocrine	5 (1.3)	4 (3.2)	1 (0.4)	
Other	21 (5.6)	10 (8.1)	11 (4.4)	
*Comorbid diseases*				
Metastatic solid tumour	82 (22.0)	18 (14.5)	64 (25.8)	0.019
Haematological malignancy	73 (19.6)	14 (11.3)	59 (23.8)	0.006
Diabetes mellitus	142 (38.2)	55 (44.4)	87 (35.1)	0.105
Chronic kidney disease	123 (33.1)	41 (33.1)	82 (33.1)	1.000
COPD	54 (14.5)	16 (12.9)	38 (15.3)	0.661
Congestive heart failure	123 (33.1)	48 (38.7)	75 (30.2)	0.129
*Severity scores*				
SOFA score	9 (6–12)	7 (4–10)	11 (8–13)	<0.001
Age-adjusted Charlson Comorbidity Index	7 (5–9)	7 (5–9)	7 (5–9)	0.098
*Laboratory in the first 24 h*				
Haemoglobin, g/dL	9.4 (8.3–11.0)	9.4 (8.1–10.9)	9.4 (8.4–11.0)	0.366
Platelets, ×10^9^/L	157.5 (68.8–248.0)	168.0 (95.0–252.0)	138.5 (58.8–240.8)	0.065
Lactate, mmol/L	2.0 (1.4–3.2)	1.8 (1.3–2.4)	2.3 (1.5–3.6)	<0.001
CRP, mg/L	115 (54–197)	78 (33–150)	144 (68–220)	<0.001
Procalcitonin, ng/mL	1.6 (0.4–8.4)	0.6 (0.2–2.6)	2.5 (0.7–12.1)	<0.001
Creatinine, mg/dL	1.6 (0.9–2.7)	1.4 (0.9–2.4)	1.7 (1.0–2.9)	0.071
Total bilirubin, mg/dL	0.8 (0.4–1.7)	0.6 (0.4–1.1)	0.8 (0.5–2.0)	0.003
Albumin, g/dL	2.7 (2.2–3.0)	2.8 (2.4–3.2)	2.5 (2.2–3.0)	<0.001
*Albumin-based ratios*				
CAR (CRP/albumin)	46.6 (18.3–79.1)	27.6 (12.1–57.7)	55.1 (25.8–88.6)	<0.001
PAR (procalcitonin/albumin)	0.6 (0.1–3.5)	0.2 (0.1–1.1)	1.0 (0.3–4.8)	<0.001
LAR (lactate/albumin)	0.8 (0.5–1.3)	0.7 (0.5–0.9)	0.9 (0.6–1.4)	<0.001
*Organ support, n (%)*				
Invasive mechanical ventilation	269 (72.3)	46 (37.1)	223 (89.9)	<0.001
Vasopressor support	286 (76.9)	57 (46.0)	229 (92.3)	<0.001
Renal replacement therapy	150 (40.3)	34 (27.4)	116 (46.8)	<0.001
*Outcomes*				
ICU stay, days	8 (5–15)	5 (4–10)	10 (6–16)	<0.001
Hospital stay, days	20 (11–32)	19 (13–28)	20 (11–34)	0.949
In-hospital death, *n* (%)	268 (72.0)	20 (16.1)	248 (100.0)	–

Data are median (IQR) or *n* (%); groups were compared with the Mann–Whitney U test or the chi-square test. Italicised rows are group headings and do not contain data. Laboratory values are the worst values recorded within the first 24 h. CAR, C-reactive protein-to-albumin ratio; COPD, chronic obstructive pulmonary disease; CRP, C-reactive protein; ICU, intensive care unit; IQR, interquartile range; LAR, lactate-to-albumin ratio; PAR, procalcitonin-to-albumin ratio; SOFA, Sequential Organ Failure Assessment.

**Table 2:** Discrimination of individual predictors for ICU mortality.

Predictor	AUC (95% CI)
SOFA score	0.730 (0.677–0.782)
Age-adjusted Charlson Comorbidity Index	0.552 (0.491–0.614)
Albumin	0.633 (0.573–0.692)
CRP	0.656 (0.597–0.715)
Procalcitonin	0.681 (0.622–0.740)
Lactate	0.611 (0.552–0.669)
CAR	0.671 (0.614–0.729)
PAR	0.689 (0.630–0.747)
LAR	0.642 (0.585–0.700)

AUCs were estimated by the DeLong method; albumin was entered inversely. Pairwise comparison of the ratio AUCs: CAR versus PAR, *p* = 0.561; CAR versus LAR, *p* = 0.415; PAR versus LAR, *p* = 0.184. AUC, area under the receiver-operating-characteristic curve; CAR, C-reactive protein-to-albumin ratio; CI, confidence interval; LAR, lactate-to-albumin ratio; PAR, procalcitonin-to-albumin ratio; SOFA, Sequential Organ Failure Assessment.

**Table 3:** Incremental prognostic value of CAR, PAR and LAR added to the base model.

Model	AUC (95% CI)	OR per SD (95% CI)	ΔAUC	NRI (95% CI)	IDI (95% CI)	HL *p*
Base + CAR	0.765 (0.715–0.815)	1.65 (1.29–2.13)	+0.030	+0.39 (+0.17–+0.61)	+0.042 (+0.013–+0.091)	0.69
Base + PAR	0.751 (0.700–0.802)	1.50 (1.15–1.95)	+0.016	+0.28 (+0.06–+0.58)	+0.023 (+0.002–+0.065)	0.53
Base + LAR	0.742 (0.690–0.793)	1.28 (1.00–1.64)	+0.006	+0.22 (–0.02–+0.48)	+0.009 (+0.000–+0.031)	0.89
Base + CAR + PAR	0.768 (0.718–0.817)	n/a	+0.032	+0.47 (+0.21–+0.68)	+0.045 (+0.016–+0.097)	0.02
*CAR (adjusted for PAR)*		1.52 (1.15–2.03)				
*PAR (adjusted for CAR)*		1.20 (0.89–1.63)				

Base model = SOFA, sex and age-adjusted CCI. OR per 1 SD of the log-transformed ratio. ΔAUC is relative to the base model (DeLong *p*: CAR, *p* = 0.024; PAR, *p* = 0.168; LAR, *p* = 0.360; CAR + PAR, *p* = 0.020). NRI (category-free) and IDI are shown with bootstrap 95% CIs from 1000 resamples. Indented italicised rows show each ratio adjusted for the other within the combined model. n/a, not applicable: the combined model contains two ratios, whose individual odds ratios are shown in the indented rows. CAR, C-reactive protein-to-albumin ratio; HL, Hosmer–Lemeshow calibration *p*; IDI, integrated discrimination improvement; LAR, lactate-to-albumin ratio; NRI, net reclassification improvement; OR, odds ratio; PAR, procalcitonin-to-albumin ratio; SD, standard deviation.

**Table 4:** Exploratory cut-offs and classification performance of CAR, PAR and LAR for ICU mortality.

Marker	Cut-Off	Sensitivity (95% CI)	Specificity (95% CI)	PPV	NPV	Mortality≥ vs. <Cut-Off
CAR	45.9	0.60 (0.54–0.66)	0.68 (0.59–0.75)	0.79	0.46	79% vs. 54%
PAR	0.48	0.64 (0.58–0.69)	0.68 (0.59–0.75)	0.80	0.48	80% vs. 52%
LAR	0.93	0.51 (0.45–0.57)	0.78 (0.70–0.85)	0.82	0.44	82% vs. 56%

Youden-derived cut-offs; apparent (in-sample) performance, exploratory. Sensitivity and specificity are shown with Wilson 95% CIs; PPV and NPV reflect the 66.7% cohort mortality. CAR, C-reactive protein-to-albumin ratio; LAR, lactate-to-albumin ratio; NPV, negative predictive value; PAR, procalcitonin-to-albumin ratio; PPV, positive predictive value.

**Table 5:** Marker levels by survival and discrimination of CAR, PAR and LAR in septic versus non-septic patients.

Marker	Survivors	Non-Survivors	*p*	AUC (95% CI)
*Septic patients (n = 116, mortality 74%)*				
CAR	56.9 (21.4–101.4)	65.4 (41.1–92.5)	0.490	0.543 (0.416–0.669)
PAR	0.94 (0.28–4.86)	2.11 (0.72–12.62)	0.075	0.610 (0.484–0.736)
LAR	0.90 (0.48–1.52)	1.00 (0.67–1.57)	0.240	0.572 (0.450–0.695)
*Non-septic patients (n = 256, mortality 63%)*				
CAR	22.4 (7.8–43.3)	50.2 (20.3–86.1)	<0.001	0.698 (0.633–0.763)
PAR	0.14 (0.05–0.47)	0.61 (0.19–3.04)	<0.001	0.702 (0.634–0.769)
LAR	0.61 (0.43–0.85)	0.86 (0.55–1.40)	<0.001	0.652 (0.585–0.718)

Data are median (IQR); survivors were compared with non-survivors within each subgroup using the Mann–Whitney U test, and AUCs are shown with DeLong 95% CIs. Italicised rows indicate subgroup headings and report subgroup size and mortality. CAR, C-reactive protein-to-albumin ratio; IQR, interquartile range; LAR, lactate-to-albumin ratio; PAR, procalcitonin-to-albumin ratio.

**Table 6:** Comparison of the analysis cohort with admissions excluded for an incomplete first 24 h work-up and with admissions that did not reach the 24 h landmark.

Variable	Analysis Cohort (*n* = 372)	Excluded, Incomplete Work-Up (*n* = 626)	Excluded, <24 h Stay (*n* = 304)	*p* *
Age, years	70 (59–77)	70 (60–79)	70 (59–77)	0.426
Male sex, *n* (%)	200 (53.8)	378 (60.4)	174 (57.2)	0.047
SOFA score	9 (6–12)	8 (4–11)	7 (4–12)	<0.001
Charlson Comorbidity Index, unadjusted	4 (3–6)	4 (2–6)	4 (2–6)	0.043
Sepsis on admission, *n* (%)	116 (31.2)	350 (55.9)	137 (45.1)	<0.001
Respiratory failure on admission, *n* (%)	158 (42.5)	224 (35.8)	131 (43.1)	<0.001
Invasive mechanical ventilation, *n* (%)	269 (72.3)	403 (64.4)	142 (46.7)	0.012
Vasopressor support, *n* (%)	286 (76.9)	383 (61.2)	145 (47.7)	<0.001
Renal replacement therapy, *n* (%)	150 (40.3)	199 (31.8)	73 (24.0)	0.008
ICU stay, days	8 (5–15)	7 (4–13)	0 (0–0)	<0.001
ICU mortality, *n* (%)	248 (66.7)	388 (62.0)	158 (52.0)	0.155

Data are median (IQR) or *n* (%). * Analysis cohort versus admissions excluded for an incomplete first 24 h work-up, by Mann–Whitney U test or chi-square test; admissions that did not reach the landmark are shown for completeness and were not formally compared. The Charlson Comorbidity Index is shown unadjusted for age so that all three groups are on the same scale. ICU, intensive care unit; IQR, interquartile range; SOFA, Sequential Organ Failure Assessment.

## Data Availability

The data presented in this study are available on request from the corresponding author. The data are not publicly available owing to privacy and ethical restrictions.

## Abbreviations

The following abbreviations are used in this manuscript:

AUC	area under the receiver-operating-characteristic curve
CAR	C-reactive protein-to-albumin ratio
CCI	Charlson Comorbidity Index
CI	confidence interval
CRP	C-reactive protein
ICU	intensive care unit
IDI	integrated discrimination improvement
IQR	interquartile range
LAR	lactate-to-albumin ratio
NRI	net reclassification improvement
PAR	procalcitonin-to-albumin ratio
PCT	procalcitonin
SD	standard deviation
SOFA	Sequential Organ Failure Assessment

## Appendix A

Table A1 gives the complete specification of every logistic regression model reported in this manuscript, so that the models can be applied or evaluated in an external cohort. Table A2 gives the model of the probability of inclusion from which the selection weights were derived, and Table A3 the covariate balance those weights achieved. Each ratio was natural-log-transformed and standardised as z = (ln X minus the mean of ln X) divided by the standard deviation of ln X. In this cohort the mean and standard deviation of ln CAR were 3.558 and 1.170, of ln PAR −0.311 and 2.184, and of ln LAR −0.222 and 0.817.

**Table A1:** Complete specification of the logistic regression models for ICU mortality.

Model and Variable	β (SE)	OR (95% CI)	*p*
*Base model*			
Intercept	−1.804 (0.441)	–	<0.001
SOFA score, per point	+0.242 (0.035)	1.27 (1.19–1.36)	<0.001
Male sex	−0.188 (0.242)	0.83 (0.52–1.33)	0.437
Age-adjusted CCI, per point	+0.069 (0.040)	1.07 (0.99–1.16)	0.084
*Base + CAR*			
Intercept	−1.421 (0.451)	–	0.002
SOFA score, per point	+0.225 (0.036)	1.25 (1.17–1.34)	<0.001
Male sex	−0.212 (0.248)	0.81 (0.50–1.32)	0.394
Age-adjusted CCI, per point	+0.043 (0.040)	1.04 (0.96–1.13)	0.291
log CAR, per 1 SD	+0.504 (0.128)	1.65 (1.29–2.13)	<0.001
*Base + PAR*			
Intercept	−1.410 (0.461)	–	0.002
SOFA score, per point	+0.202 (0.037)	1.22 (1.14–1.32)	<0.001
Male sex	−0.160 (0.245)	0.85 (0.53–1.38)	0.514
Age-adjusted CCI, per point	+0.066 (0.040)	1.07 (0.99–1.16)	0.096
log PAR, per 1 SD	+0.403 (0.135)	1.50 (1.15–1.95)	0.003
*Base + LAR*			
Intercept	−1.650 (0.447)	–	<0.001
SOFA score, per point	+0.229 (0.035)	1.26 (1.17–1.35)	<0.001
Male sex	−0.180 (0.243)	0.84 (0.52–1.35)	0.459
Age-adjusted CCI, per point	+0.064 (0.040)	1.07 (0.99–1.15)	0.107
log LAR, per 1 SD	+0.249 (0.126)	1.28 (1.00–1.64)	0.048
*Base + CAR + PAR*			
Intercept	−1.307 (0.462)	–	0.005
SOFA score, per point	+0.209 (0.038)	1.23 (1.14–1.33)	<0.001
Male sex	−0.197 (0.249)	0.82 (0.50–1.34)	0.428
Age-adjusted CCI, per point	+0.047 (0.041)	1.05 (0.97–1.13)	0.250
log CAR, per 1 SD	+0.421 (0.145)	1.52 (1.15–2.03)	0.004
log PAR, per 1 SD	+0.185 (0.154)	1.20 (0.89–1.63)	0.229

Coefficients are on the log-odds scale, with standard errors in parentheses; odds ratios are shown with Wald 95% confidence intervals. Italicised rows indicate model headings and do not contain data. The base model comprises the SOFA score, sex and the age-adjusted CCI. Each ratio is entered as the standardised natural logarithm, so its odds ratio corresponds to a 1 SD increase. CAR, C-reactive protein-to-albumin ratio; CCI, Charlson Comorbidity Index; CI, confidence interval; LAR, lactate-to-albumin ratio; OR, odds ratio; PAR, procalcitonin-to-albumin ratio; SD, standard deviation; SOFA, Sequential Organ Failure Assessment.

**Table A2:** Logistic model of the probability of inclusion in the analysis cohort among the 998 admissions reaching the 24 h landmark.

Variable	β (SE)	OR (95% CI)	*p*
Intercept	−1.234 (0.378)	–	0.001
Age, per year	−0.004 (0.005)	1.00 (0.99–1.01)	0.364
Male sex	−0.339 (0.147)	0.71 (0.53–0.95)	0.021
SOFA score, per point	+0.086 (0.021)	1.09 (1.05–1.14)	<0.001
Charlson index (unadjusted), per point	+0.038 (0.029)	1.04 (0.98–1.10)	0.184
Invasive mechanical ventilation	−0.045 (0.189)	0.96 (0.66–1.38)	0.813
Vasopressor support	+0.918 (0.200)	2.50 (1.69–3.70)	<0.001
Renal replacement therapy	+0.176 (0.157)	1.19 (0.88–1.62)	0.262
*Admission category (reference: respiratory failure)*			
Cardiovascular	+1.700 (0.656)	5.48 (1.51–19.81)	0.010
Gastrointestinal/hepatic	+0.958 (0.318)	2.61 (1.40–4.87)	0.003
Metabolic/endocrine	+1.105 (0.767)	3.02 (0.67–13.58)	0.150
Neurologic	+0.590 (0.555)	1.80 (0.61–5.35)	0.287
Other	+1.262 (0.435)	3.53 (1.50–8.29)	0.004
Postoperative/trauma	+0.762 (0.497)	2.14 (0.81–5.67)	0.125
Sepsis	−1.256 (0.171)	0.28 (0.20–0.40)	<0.001

Coefficients are on the log-odds scale with standard errors in parentheses; odds ratios are shown with Wald 95% confidence intervals. Italicised text indicates the categorical variable heading; respiratory failure is the reference category. The outcome is inclusion in the analysis cohort (*n* = 372) versus exclusion for an incomplete first 24 h work-up (*n* = 626). The model discriminated with an area under the curve of 0.737. Stabilised inverse-probability-of-selection weights were derived as the reciprocal of the fitted probability. CI, confidence interval; OR, odds ratio; SE, standard error; SOFA, Sequential Organ Failure Assessment.

**Table A3:** Balance between the analysis cohort and the full landmark population before and after inverse-probability-of-selection weighting.

Covariate	SMD Before Weighting	SMD After Weighting	Balanced (|SMD| < 0.10)
Age	−0.043	−0.048	Yes
Male sex	−0.084	+0.030	Yes
SOFA score	+0.218	−0.022	Yes
Charlson index (unadjusted)	+0.095	+0.008	Yes
Invasive mechanical ventilation	+0.108	−0.114	No
Vasopressor support	+0.220	−0.053	Yes
Renal replacement therapy	+0.111	+0.016	Yes
*Admission category*			
Cardiovascular	+0.150	−0.005	Yes
Gastrointestinal/hepatic	+0.149	−0.003	Yes
Metabolic/endocrine	+0.053	−0.003	Yes
Neurologic	+0.048	−0.004	Yes
Other	+0.130	−0.003	Yes
Postoperative/trauma	+0.052	−0.013	Yes
Respiratory failure	+0.086	−0.092	Yes
Sepsis	−0.322	+0.097	Yes

Standardised mean differences (SMD) compare the analysis cohort (*n* = 372), unweighted and weighted, with all 998 admissions reaching the 24 h landmark. Italicised text indicates the categorical variable heading and does not contain data; admission category entries are indicator variables. An absolute SMD below 0.10 was taken as adequate balance. SOFA, Sequential Organ Failure Assessment.
